# Postpartum Ischemic Stroke: A Rare Case

**DOI:** 10.7759/cureus.9975

**Published:** 2020-08-23

**Authors:** Umar Farooque, Omer Cheema, Sundas Karimi, Bharat Pillai, Muhammad Talha Liaquat

**Affiliations:** 1 Neurology, Dow University of Health Sciences, Karachi, PAK; 2 Internal Medicine, Dow University of Health Sciences, Karachi, PAK; 3 General Surgery, Combined Military Hospital, Karachi, PAK; 4 Neurology, Amrita Institute of Medical Sciences, Kochi, IND; 5 Internal Medicine, King Edward Medical University, Lahore, PAK

**Keywords:** postpartum, ischemic stroke, pregnancy, humans, cesarean section, complications

## Abstract

The risk of stroke is increased during pregnancy and the postpartum period. It can lead to significant maternal morbidity and mortality. The physiologically mediated hemodynamic changes in circulation and vascular tissue, and the increased coagulability account for this increased risk of stroke. Pregnancy-related strokes can be hemorrhagic or ischemic. We present a rare case of postpartum ischemic stroke. A 25-year-old female with no known comorbidities and a history of cesarean section 10 days back presented with a right-sided weakness and sensory loss for one day. An MRI of the head revealed a large area of restricted diffusion on diffusion-weighted 1 (DW1) image in the left parietal region with comparable low signals on apparent diffusion coefficient (ADC) map and a small area of blooming suggesting hemorrhage on susceptibility-weighted 1 (SW1) image. This area appeared hypointense on T1 and hyperintense on fluid-attenuated inversion recovery (FLAIR) and T2 images. These findings suggested acute ischemic infarction. She was started on antiplatelet therapy, and subsequently, her weakness improved. She was discharged upon improvement in her symptoms and was asked to follow up in the outpatient department. Numerous studies have shown an increased risk of ischemic stroke in the immediate postpartum period in women who undergo a cesarean section. Thus, we conclude that clinicians should be aware of this complication and high-risk patients should be identified and monitored more aggressively in their immediate postpartum period.

## Introduction

Pregnancy-associated stroke is an important cause of maternal morbidity and mortality, which creates significant diagnostic and therapeutic challenges. The incidence is approximately between 11 and 26 per 100,000 deliveries, with the risk increasing in the postpartum period [1,2]. According to one study, the risk of postpartum stroke readmission was highest within 10 days of hospital discharge, with 58.4% cases occurring within 10 days [3]. Timely interventions and multidisciplinary collaboration among health care professionals are imperative in diagnosing and effectively managing such cases. We report one such case from Nawabshah, Pakistan.

## Case presentation

A 25-year-old female, para two and gravida two, with no known comorbidities, and history of cesarean section 10 days back, presented to the emergency department with a right-sided upper and lower limb weakness and sensory loss for one day. According to the patient, she was in her usual state of health when she suddenly developed right-sided weakness and sensory loss that was not associated with fits, fever, or headache. There was no family history of early-onset stroke.

On examination, she was of average height and build, well oriented, and lying comfortably in the bed. Her temperature was 98.6^o^F, blood pressure was 110/70 mmHg, pulse was 90 beats per minute, and respiratory rate was 20 breaths per minute. On systemic examination her Glasgow Coma Scale (GCS) was 15/15; however, the power of the right upper and lower limb was 0/5 and upgoing right plantar. She also had a loss of all sensations in the right upper and lower limb. Other systems were unremarkable on examination.

**Figure 1:**
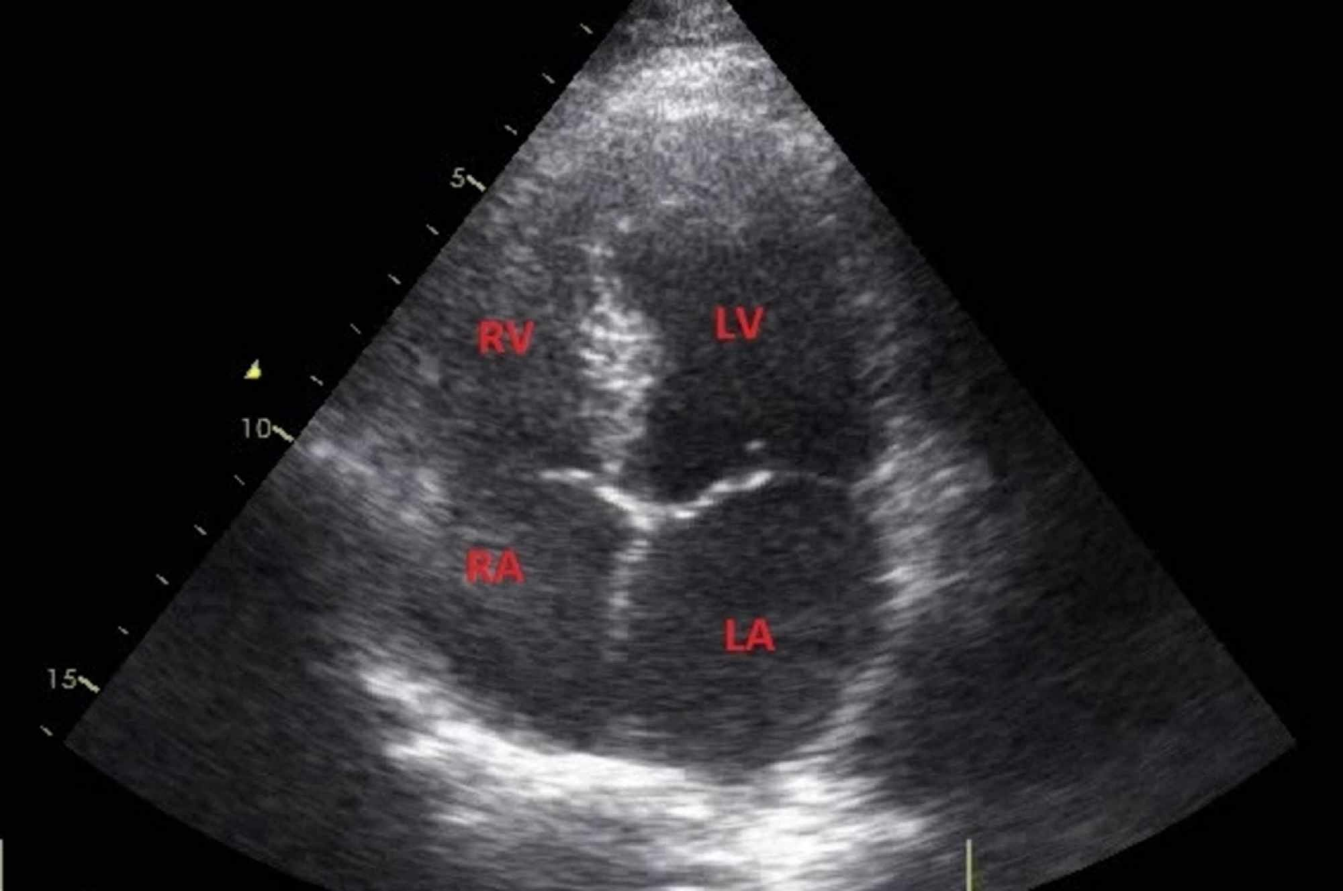
Transthoracic echocardiogram This is an apical four-chamber view of a normal transthoracic echocardiogram of the heart RA, right atrium; RV, right ventricle; LA, left atrium; LV, left ventricle

Laboratory tests revealed the following: hemoglobin 13 g/dL, white cell count 8.0 x 10^9^ cells/L, and platelets 188 x 10^9^ cells/L. Random blood sugar (RBS), lipid profile, liver function test (LFT), urea, creatinine, and electrolytes were normal. Hepatitis B surface antigen (HBsAg) and anti-hepatitis C virus antibody (anti-HCV) were non-reactive. Thyroid-stimulating hormone (TSH), vitamin B12, and hypercoagulability workup were within normal limits. Transthoracic echocardiography was unremarkable with good systolic function and no evidence of thrombus, patent foramen ovale, or atrial septal defect was found (Figure 1).

**Figure 2:**
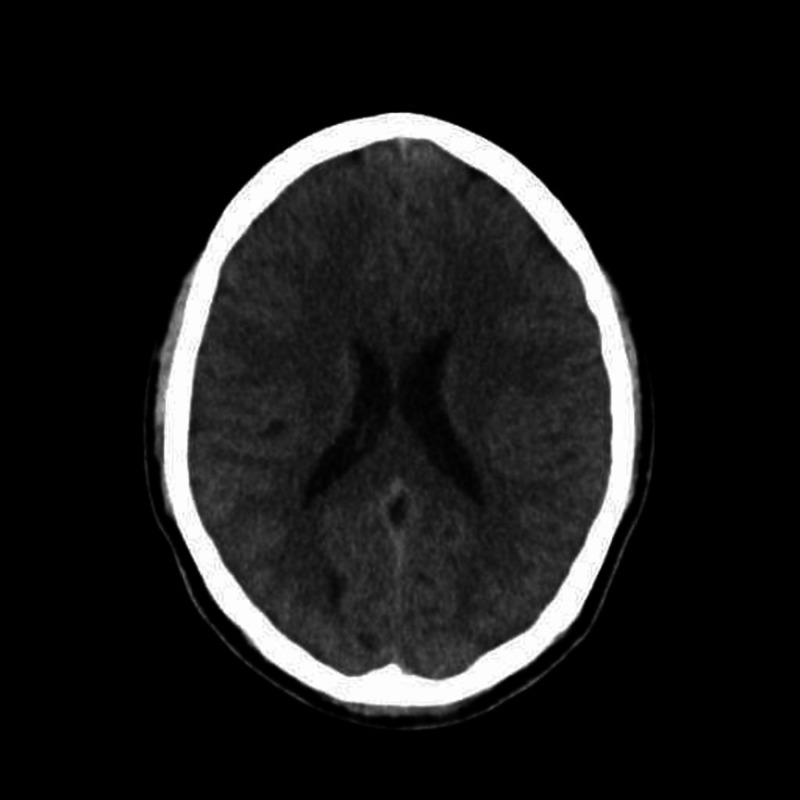
CT scan of head in axial view This is a normal CT scan of the head.

CT scan of the head was normal (Figure 2).

**Figure 3:**
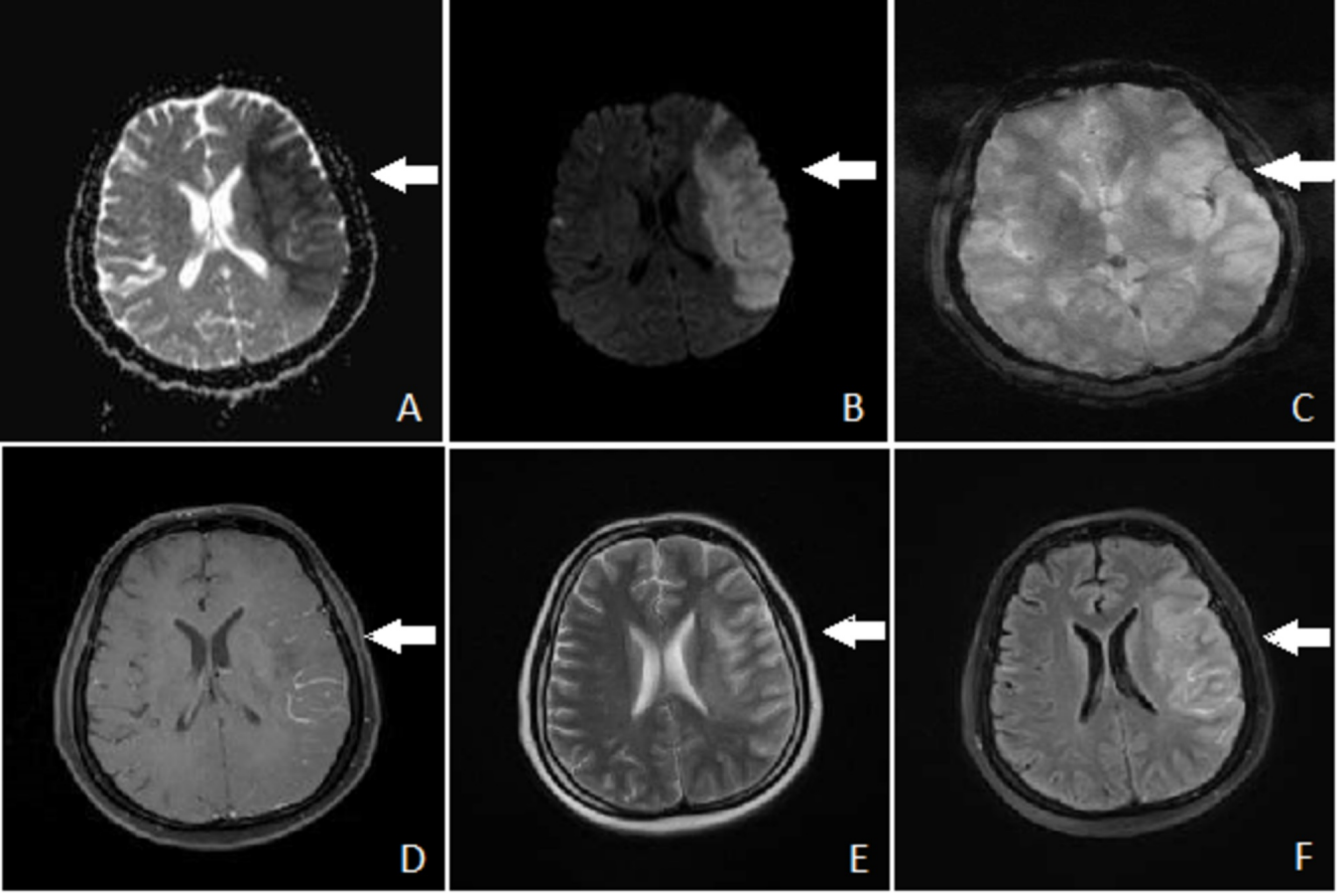
MRI scan of head in axial view (A) Apparent diffusion coefficient (ADC) scan showing low signals in a large area of left parietal region. (B) Diffusion weighted 1 (DW1) scan showing a large area of restricted diffusion in left parietal region. (C) Susceptibility weighted 1 (SW1) scan showing a small area of blooming in left parietal region suggesting hemorrhage. (D) Contrast-enhanced T1 scan showing subtle gyriform enhancement in left parietal region. (E) T2 scan showing abnormal high signals in left parietal region. (F) Fluid-attenuated inversion recovery (FLAIR) scan showing abnormal high signals in left parietal region with perifocal edema obscuring grey white interface.

MRI scan of the head was done that showed a large area of restricted diffusion on diffusion-weighted 1 (DW1) image in the left parietal region with corresponding low signals on apparent diffusion coefficient (ADC) map (Figure 3A, 3B). Susceptibility-weighted 1 (SW1) image showed a small area of blooming in the left parietal region suggesting hemorrhage (Figure 3C). This area appeared low on T1 and high on fluid-attenuated inversion recovery (FLAIR) and T2 images (Figure 3D-3F). These findings were suggestive of acute ischemic infarction.

The patient was started on aspirin 300 mg once daily, clopidogrel 75 mg once daily, and simvastatin 40 mg once daily. Her power in the right upper and lower limb gradually improved to 3/5. She was discharged after improvement in her symptoms and was asked to follow up in the outpatient department.

## Discussion

The purpose of this case report is to draw attention to an increased risk of stroke in the postpartum period in women who undergo a cesarean section.

Pregnant women are at an increased risk for thromboembolic diseases. This is due to the physiologic changes in circulation and increased coagulability, which predisposes them to stroke and other thromboembolic complications [4]. Many pre-existing medical conditions increase the risk of stroke including hypertension, diabetes mellitus, heart disease, migraines, antiphospholipid syndrome, sickle cell disease, and thrombophilia [2]. Several pregnancy-related complications such as pre-eclampsia, eclampsia, postpartum hemorrhage, amniotic fluid embolism, and peripartum cardiomyopathy have been linked to increased incidence of stroke as well.

Our case report concerns with a rare presentation of a postpartum ischemic stroke occurring after the cesarean section. The risk of postpartum stroke is increased during the first year after delivery according to Cheng et al., with 58.4% cases occurring within 10 days postpartum [3,5]. The exact mechanism for increased risk in the postpartum period is unknown, but a rise in blood pressure on three to five days due to fluid shift and impaired cerebral autoregulation can increase the chances of stroke [6]. Numerous studies have identified the cesarean section as a potential risk factor for ischemic stroke. Studies by Witlin et al. and Lanska and Kryscio have identified an association between postpartum stroke and cesarean section and hypertensive disorders of pregnancy [7,8]. Our patient had no other major risk factors besides the recent cesarean section, which may have been the reason for her stroke.

Timely assessment and management of postpartum stroke are of paramount importance as it carries a mortality rate of 2%-10% [9]. The management of postpartum stroke is challenging because of pregnancy being an exclusion criterion for reperfusion therapy. Although intravenous thrombolysis has shown to reduce morbidity in non-pregnant women, its safety and efficacy in the early postpartum period are not well established [9]. The use of thrombolytics, as suggested by Akazawa and Nishida, was associated with a high risk of bleeding after the cesarean section in comparison with vaginal deliveries; thus, its use was worth avoiding [10]. Symptoms of stroke can be overlooked in the postpartum period as they can mimic those seen in pre-eclampsia and eclampsia. Thus, patients delay seeking medical care, which can adversely affect the outcome.

## Conclusions

Postpartum ischemic stroke is rare but can occur in the postpartum period especially after cesarean section. Due to the above-mentioned challenges associated with its diagnosis and management, high-risk women should be identified and monitored in the postpartum period for thrombotic complications. Protocols for secondary prevention such as blood pressure and diabetes management should be implemented, and extending the recommended duration of prophylactic anticoagulant therapy may be considered after careful assessment of risk to benefit ratio.
